# Persistent Response to a Combination Treatment Featuring a Targeted Agent and an Immune Checkpoint Inhibitor in a Patient With Collecting Duct Renal Carcinoma: A Case Report and Literature Review

**DOI:** 10.3389/fonc.2021.764352

**Published:** 2021-11-08

**Authors:** Weimin Zhou, Ji Huang, Qiuming He, Qingfeng Luo, Xiaofang Zhang, Xuewei Tao, Hanzhi Dong, Xinhua Tu

**Affiliations:** ^1^ Department of Urology, Jiangxi Cancer Hospital of Nanchang University, Jiangxi Cancer Center, Nanchang, China; ^2^ Department of Pathology, Jiangxi Cancer Hospital of Nanchang University, Jiangxi Cancer Center, Nanchang, China; ^3^ Department of Radiology, Jiangxi Cancer Hospital of Nanchang University, Jiangxi Cancer Center, Nanchang, China; ^4^ Department of Internal Medical Oncology, Jiangxi Cancer Hospital of Nanchang University, Jiangxi Cancer Center, Nanchang, China

**Keywords:** collecting duct carcinoma, kidney cancer, immunotherapy, targeted therapy, immune checkpoint inhibitor

## Abstract

Collecting duct carcinoma (CDC) is a rare and highly aggressive subtype of kidney cancer that is associated with a poor prognosis. At present, there is no effective treatment for CDC. Herein, we report a case of metastatic CDC treated with a combination of a tyrosine kinase inhibitor and an immune checkpoint inhibitor. A 67-year-old male was diagnosed with CDC with lung and bone metastasis. Pazopanib and camrelizumab were administered after cytoreductive nephrectomy. The patient achieved a partial response after one cycle of treatment; however, he then experienced serious drug-induced hepatic injury. Therefore, we discontinued camrelizumab and administered monotherapy with pazopanib. Three months later, the cancer had progressed and axitinib and sintilimab were administered. The patient achieved a partial response, accompanied by the complete disappearance of the metastatic lesion in the lung. The patient had an excellent physical status after 11 months. This is the first reported case of metastatic CDC successfully treated with a combination of a tyrosine kinase inhibitor and an immune checkpoint inhibitor. This form of combination treatment may be an effective option for treating metastatic CDC.

## Introduction

Renal collecting duct carcinoma (CDC), also referred to as Bellini duct carcinoma, is a subtype of renal cell carcinoma (RCC) with unique clinical and pathological characteristics. This is a rare condition and accounts for only 0.4–2.0% of RCC cases (1–3). CDC originates from distal convoluted tubules of the kidney (3) and is characterized by high rates of invasiveness and early metastasis, as well as a poor prognosis. According to a previous study, >70% of patients with CDC had distant metastasis at their initial diagnosis; their median overall survival (OS) was approximately 13 months (4). The biological characteristics of CDC are similar to those of urothelial carcinoma (5).

Thus far, there is a lack of effective treatment options for metastatic CDC (mCDC) (6). The combination of gemcitabine with platinum salt chemotherapy showed efficacy in a previous study involving 23 cases of mCDC (7). In this previous cohort, one complete and five partial responses (objective response rate: 26%) were observed; however, the median progression-free survival (PFS) and OS were only 7.1 and 10.5 months, respectively (7). Nevertheless, platinum-based chemotherapy is considered a standard therapeutic regimen for mCDC. Targeted agents (e.g., sorafenib, temsirolimus, sunitinib, and cabozantinib) have shown activity in certain mCDC cases (8, 9). Although immune checkpoint inhibitors (ICIs) may be effective against some mCDCs, the benefit of monotherapy with these agents is limited (10). In earlier studies, three cases of mCDC receiving a combination immunotherapy of nivolumab, a programmed cell death 1 (PD-1) antibody, and ipilimumab, a cytotoxic T-lymphocyte associated protein 4 (CTLA4) antibody, the first-line therapy for clear cell RCC (ccRCC), all achieved excellent disease control (11, 12).

The combination of immunotherapy and targeted therapy plays a joint role in the treatment of advanced ccRCC and is recommended as a first-line therapy. However, the efficacy of combination immunotherapy and targeted therapy against CDC remains unclear. Herein, we report a case that demonstrated the efficacy of the combination of targeted therapy and PD-1 antibody against mCDC.

## Case Presentation

**Figure 1:**
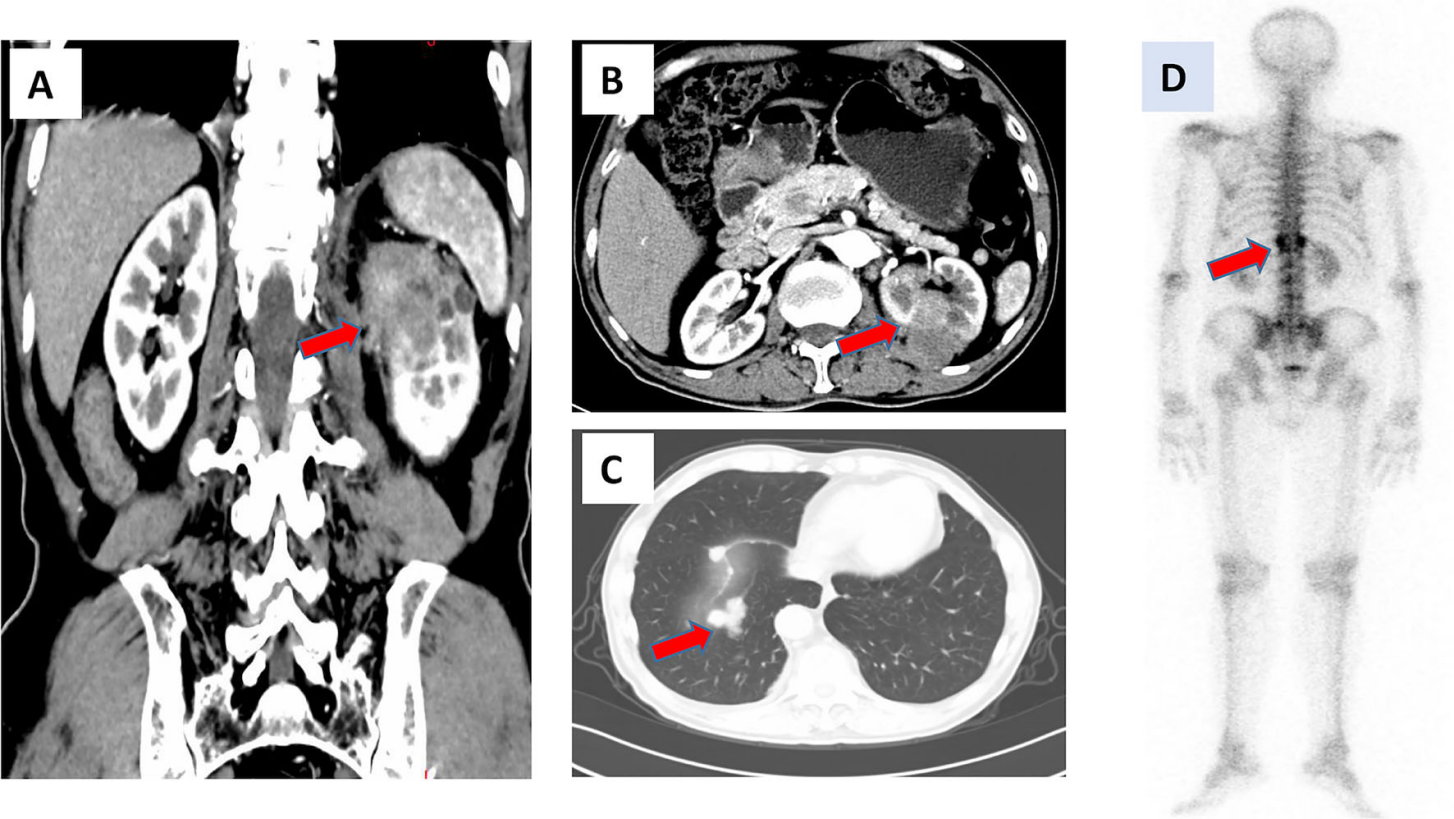
Radiological examination at initial visit. **(A, B)** Contrast-enhanced CT scan of the abdomen showing a malignant mass in the left kidney. **(C)** Chest CT scan showing metastatic nodules in the right lower lung. **(D)** Whole body bone scan showing abnormal T12 vertebration. CT, computed tomography.

In October 2019, a 67-year-old man was admitted to our hospital due to left flank pain. He was initially diagnosed with a left renal tumor based on ultrasound examination. The patient had a history of controlled hypertension for approximately 10 years, but no personal or family history of other systemic disorders. Contrast-enhanced computed tomography (CT) revealed the presence of a malignant mass (5.2 cm × 4.3 cm) (**Figures 1A, B**). Chest CT revealed multiple nodules in the right lower lung, indicating metastasis (**Figure 1C**). Whole-body bone scanning by emission CT suggested vertebral (T12) metastasis (**Figure 1D**). The Eastern Cooperative Oncology Group score of the patient was 1. Routine blood and blood biochemistry tests did not yield abnormal findings. Based on these findings, the patient was diagnosed with advanced left renal carcinoma graded cT2N×M1.

**Figure 2:**
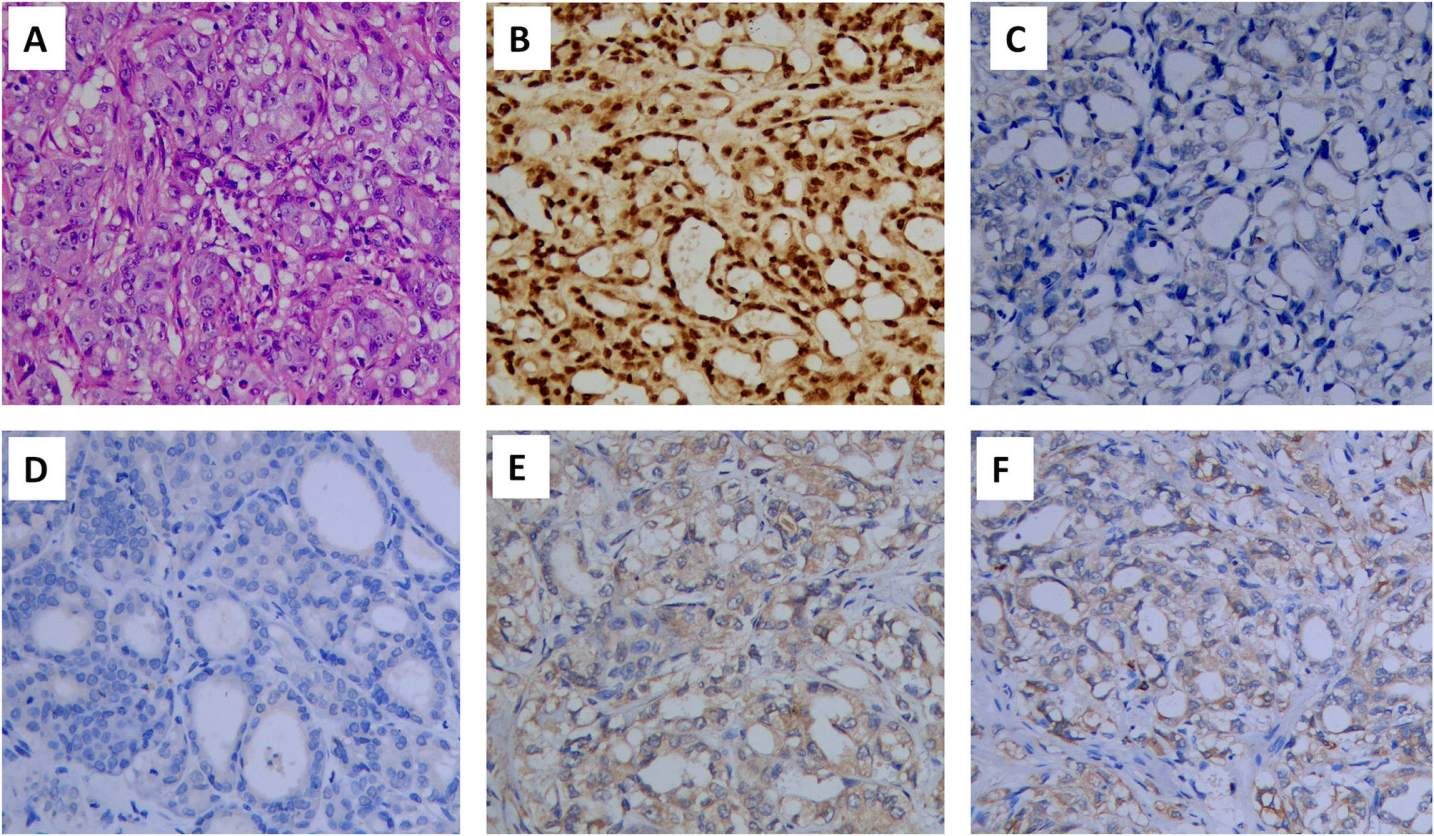
Hematoxylin and eosin (HE) staining and immunohistochemical staining of the renal tumor (×200): **(A)** HE staining, **(B)** PAX8 (positive), **(C)** p63 (negative), **(D)** CD10(negative), **(E)** vinculin (positive) and **(F)** PD-L1(positive). PAX8, paired box 8; PD-L1, programmed cell death 1 ligand 1.

**Figure 3:**
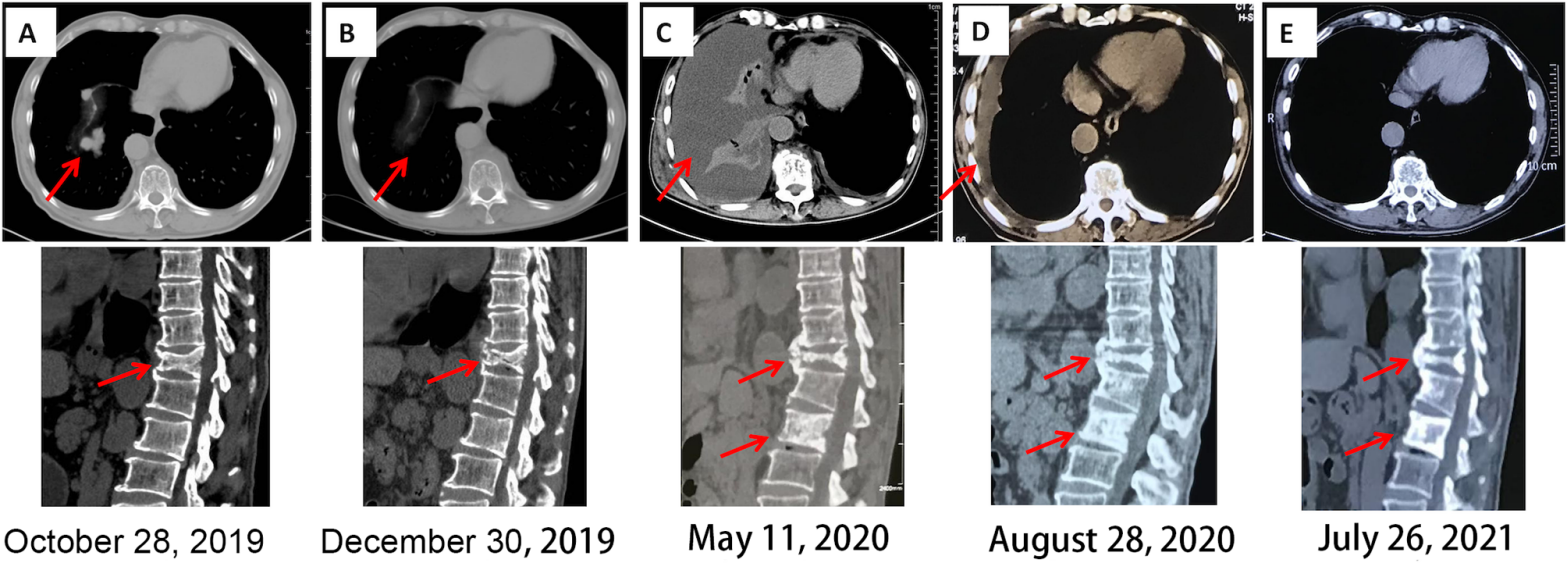
Chest and spinal CT imaging manifestations after treatment. **(A)** First diagnosis. **(B)** After one cycle of pazopanib and camrelizumab. **(C)** Discontinuation of camrelizumab and monotherapy involving pazopanib for 5 months. **(D)** Three months after the combination treatment of axitinib and sintilimab. **(E)** Fourteen months after the combination treatment of axitinib and sintilimab. CT, computed tomography.

According to the criteria established by the International Metastatic Renal Cell Carcinoma Database Consortium (IMDC), the prognostic risk associated with one risk factor was intermediate. Thus, cytoreductive nephrectomy was recommended, and pathological examination confirmed CDC (**Figure 2A**). Immunohistochemistry was used to examine the presence of several key markers: paired box 8-negative (PAX8; positive), p63 (negative), CD10 (negative), and vinculin (positive) (**Figures 2B–E**). Immunohistochemistry was positive for programmed cell death 1 ligand 1 (PD-L1) (**Figure 2F**). Next, we performed genetic profiling using a customized panel consisting of 618 genes to investigate potential actionable somatic and pathogenic germline variants (**Supplemental Table 1**). Sequencing analysis did not identify pathogenic or likely pathogenic germline variants in this sample. However, four somatic alterations with uncertain clinical significance were detected: ERBB receptor feedback inhibitor 1 (ERRFI1) S138fs; speckle type BTB/POZ protein (SPOP) S55fs; E1A binding protein p300 (EP300) S457I; and TEK receptor tyrosine kinase (TEK) R673H. However, none of these findings supported the potential response to any of the therapies approved by the Food and Drug Administration. We recommended a treatment strategy involving the combination of a targeted agent and ICI, which has shown excellent therapeutic results in the treatment of ccRCC. The patient was fully informed and aware of the off-label use of the drugs. Pazopanib (400 mg, *per os*, once daily) and the PD-1 monoclonal antibody camrelizumab (200 mg, intravenous gtt, once every 3 weeks) were administered. Following one cycle of therapy, the patient experienced a reduction in appetite and developed severe drug-induced hepatic injury. However, chest CT showed remarkable shrinkage of the metastases, indicating a partial response (**Figures 3A, B**). Subsequently, glucocorticosteroid therapy was administered. Liver function recovered three weeks later. Immunotherapy was discontinued, and monotherapy with pazopanib was initiated.

**Figure 4:**
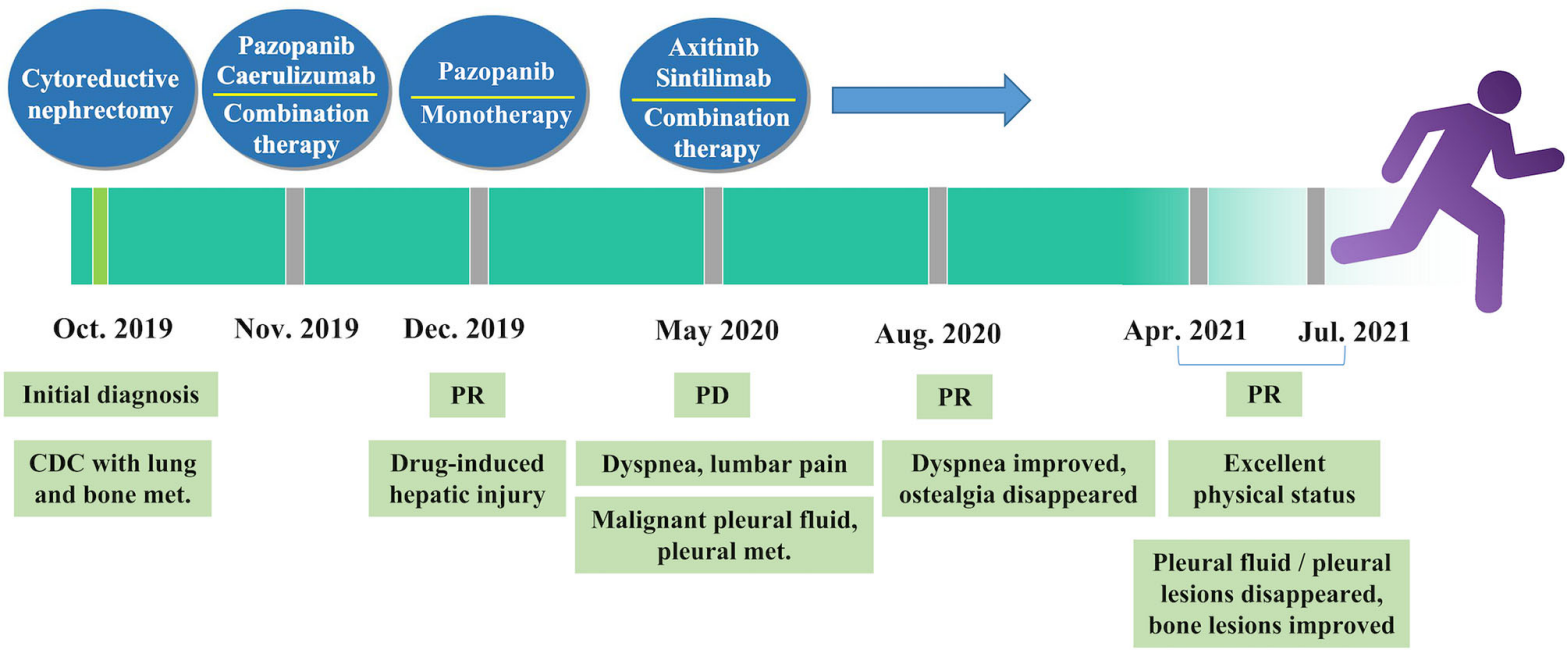
Timeline of treatment. CDC, collecting duct carcinoma; met., metastasis; PD, progressive disease; PR, partial response.

Five months later, the patient presented with cough, dyspnea, and lumbar pain. Chest CT showed malignant pleural effusion and pleural metastasis (**Figure 3C**); this was confirmed by the cytological examination of pleural effusion. Considering the response of the patient to the previous combination strategy, axitinib (5 mg, *per os*, twice daily) and the PD-1 monoclonal antibody sintilimab (200 mg, intravenous gtt, once every 3 weeks) were duly administered. The symptoms of the patient were gradually alleviated and completely disappeared after five cycles of treatment. Except for hypertension, there was no occurrence of obvious adverse reactions. Chest CT revealed a marked reduction of pleural effusion (**Figure 3D**). In April 2021, after 11 months of treatment, examinations confirmed the complete disappearance of the metastatic lesion in the lung. The last follow-up examination was performed in July 2021. Chest and abdomen CT did not reveal the presence of abnormal lesions (**Figure 3E**) and demonstrated improvement in the osteogenic structure at the site of vertebral metastasis (**Figure 3E**). The patient had an excellent physical status that was similar to that of a healthy person. Thereafter, the patient remained tumor-free for >14 months after receiving the combination therapy (**Figure 4**).

## Discussion

Research has shown that 40–70% of patients with CDC have metastatic spread at their initial presentation, and most patients die within 1–3 years from the time of primary diagnosis (13–15). To the best of our knowledge, this is the first reported case of the successful treatment of mCDC through the combination of a targeted agent and immunotherapy.

Cytotoxic chemotherapy plays an important role in the management of non-ccRCC, such as sarcomatoid RCC, renal medullary carcinoma, and CDC. In a previous multicenter prospective study, 23 patients with mCDC were treated with a combination of gemcitabine and either cisplatin or carboplatin as first-line therapy; these agents are often used as standard chemotherapy for urothelial carcinoma (7). Analysis revealed a response rate of 26% and an OS of 10.5 months, indicating that chemotherapy is an option for the treatment of CDC. Thus, the National Comprehensive Cancer Network Kidney Cancer Panel made an appropriate recommendation (16). Another study reported that the triple combination of bevacizumab, gemcitabine, and platinum salt, prolonged progression-free survival (median: 15.1 months) and OS (median: 27.8 months) in five cases of mCDC (17). However, in a prospective phase II trial of metastatic renal medullary and mCDC, this triple combination strategy was associated with low response rates and severe toxicity (18).

The role of targeted agents for the treatment of non-ccRCC warrants further investigation. Data suggested that the targeted therapies approved for ccRCC may offer benefits to patients with non-ccRCC. A previous retrospective study analyzed seven patients with mCDC who received treatment with targeted agents (i.e., sorafenib, temsirolimus, or sunitinib) as first-line therapy (8). The results showed long-lasting disease control in two cases (OS: 49 and 19 months, respectively) and early progression of disease with a very short survival period of 4 months in the remaining five patients (8). In another study, cabozantinib was used in four patients with mCDC; two patients achieved a partial response with relief times of 10 and 11 months, respectively (19). Overall, the response rates to monotherapy with a targeted agent are significantly lower for CDC when compared to ccRCC.

Currently, ICIs are an important treatment option for metastatic ccRCC. Checkpoint antibodies alter the interaction between immune cells and antigen-presenting cells, including tumor cells. These agents can augment the anti-tumor immune response and have shown promise in numerous indications. In metastatic ccRCC, monotherapy with ICI has shown limited benefit, whereas the combination of ICIs or targeted agent/ICIs has displayed encouraging efficacy (20, 21). However, there is a lack of evidence regarding the effects of combination therapy for CDC. CDC is found an immunogenic disease with a high level of immune lymphocyte infiltration, particularly in metastatic cases, suggesting that immunotherapy may be feasible for mCDC (3). In a previous study, Danno et al. (12) reported two cases of mCDC treated with combination immunotherapy consisting of nivolumab and ipilimumab. One patient achieved stable disease for 23 months, while the other achieved a partial response after four cycles of treatment. Similarly, another CDC case with multiple lymph node metastases achieved a complete response after therapy with nivolumab and ipilimumab (11).

Sintilimab and camrelizumab are both immunoglobulin G4 (IgG4) monoclonal PD-1 antibodies that are derived from humans. These antibodies block the binding of PD-1 to PD-L1 or PD-L2 (22, 23). These two drugs have been developed independently in China and have shown excellent clinical benefits in the treatment of relapsed or refractory Hodgkin’s lymphoma (24), non-small-cell lung cancer (25), and hepatocellular carcinoma (26). Pazopanib and axitinib are multitargeted tyrosine kinase inhibitors (TKIs) and have shown efficacy when combined with ICIs (21, 27). In the present case, the combination of both pazopanib/camrelizumab and axitinib/sintilimab exerted an obvious therapeutic effect. This finding suggested that the strategy of combining targeted agents and ICIs for the treatment of ccRCC may also benefit patients with CDC. However, this combination therapy may increase the incidence of adverse reactions. Although our patient achieved good results following the administration of pazopanib and camrelizumab, he subsequently developed a severe liver function reaction; his symptoms improved after the discontinuation of treatment. It is currently established that combination therapy with standard doses of pazopanib plus PD-1 antibody is associated with a high risk of hepatotoxicity (28, 29). Grade 3 transaminase elevation has been reported in up to 90% of patients (29). However, treatment with low-dose pazopanib (≤400 mg daily) may avoid the hepatoxicity. A retrospective analysis of 13 patients with metastatic RCC who received combination treatment with nivolumab and low dose pazopanib showed no hepatotoxicity, while only one patient on pazopanib starting dose 800 mg developed elevated transaminases (27). Therefore, the success of combination regimens based on ICIs and TKIs may depend on the selection of the antiangiogenic component and dosage (28).

Multidisciplinary treatment is important for advanced CDC. Cytoreductive nephrectomy is typically carried out for both diagnostic and therapeutic purposes (13). It has been shown that patients in the early stages of CDC with no lymph and distant metastasis can benefit from cytoreductive surgery (30). A retrospective analysis of 851 patients with metastatic non-ccRCC from the Surveillance, Epidemiology and End Results (SEER) database (2001–2014) revealed that, among all histological subtypes (including CDC), the cancer-specific mortality was invariably lower in patients who underwent cytoreductive nephrectomy than in those who did not (31). Recently, Sui et al. (4) conducted a retrospective study of 577 patients with CDC. Multivariate analysis revealed a survival benefit of multidisciplinary therapy with surgery plus chemotherapy and/or radiotherapy over single-mode therapy. Watanabe et al. (11) reported a case of mCDC treated with the combination of nivolumab and ipilimumab following cytoreductive nephrectomy, achieving a complete response. However, the mechanism underlying the benefits offered by cytoreductive nephrectomy in patients with mCDC has yet to be elucidated.

To our knowledge, this is the first reported patient with mCDC who was treated with a combination of a TKI and an ICI. The patient achieved a sustained response after the combination of axitinib and sintilimab following cytoreductive nephrectomy. Accumulation of additional cases and prospective studies targeting CDCs through the combination of a TKI and an ICI are warranted to improve the outcomes of this rare disease with few evidence-based treatment options. Furthermore, the use of cytoreductive nephrectomy in mCDC is worthy of reconsideration.

## Data Availability Statement

The original contributions presented in the study are included in the article/**Supplementary Material**. Further inquiries can be directed to the corresponding author.

## Ethics Statement

The studies involving human participants were reviewed and approved by Ethics Committee of Jiangxi Cancer Hospital of Nanchang University. The patients/participants provided their written informed consent to participate in this study. Written informed consent was obtained from the individual(s) for the publication of any potentially identifiable images or data included in this article.

## Author Contributions

XHT performed the surgery and participated in treatment planning. WZ collected the data and wrote the original draft of the manuscript. JH and QH prepared the draft of the manuscript. QL and XZ analyzed the pathology data. XWT analyzed the imaging data. All authors contributed to the article and approved the submitted version.

## Conflict of Interest

The authors declare that the research was conducted in the absence of any commercial or financial relationships that could be construed as a potential conflict of interest.

## Publisher’s Note

All claims expressed in this article are solely those of the authors and do not necessarily represent those of their affiliated organizations, or those of the publisher, the editors and the reviewers. Any product that may be evaluated in this article, or claim that may be made by its manufacturer, is not guaranteed or endorsed by the publisher.

## Supplementary Material

Supplementary Table 1 Genetic analysis of 618 genes related to the potential actionable somatic and pathogenic germline variants.
